# Low‐Voltage and High‐k Properties of Bilayer HZO Capacitors at the Morphotropic Phase Boundary for Next‐Generation Memory Applications

**DOI:** 10.1002/advs.202519686

**Published:** 2026-02-09

**Authors:** Junseok Kim, Hyeonjung Park, Changwoo Han, Huiseong Shin, Myeongjae Choi, Changhwan Shin

**Affiliations:** ^1^ School of Electrical Engineering Korea University Seoul Republic of Korea; ^2^ Department of Electrical and Computer Engineering Sungkyunkwan University College of Engineering Seoul Republic of Korea; ^3^ School of Semiconductor System Engineering Korea University Seoul Republic of Korea

**Keywords:** antiferroelectricity/ferroelectricity, high‐k dielectric, low‐power, morphotropic phase boundary, phase transition

## Abstract

We report the phase stabilization and capacitance enhancement of electric‐field‐treated Hf_0.5_Zr_0.5_O_2_ (HZO) bilayer capacitors near the morphotropic phase boundary (MPB). Compositional‐asymmetric bilayer structures were fabricated by atomic layer deposition, followed by post‐deposition annealing and electric‐field cycling. The applied cycling induced irreversible phase transitions between the orthorhombic (o‐) and tetragonal (t‐) phases, effectively stabilizing the MPB region. A pronounced capacitor wake‐up effect was observed, indicating self‐optimization under repeated cycling. Structural analyses confirmed distinct bilayer formation and progressive phase evolution, while electrical measurements revealed an enhanced dielectric constant and increased remanent polarization. Importantly, the optimized bilayer exhibited a maximum dielectric constant of ∼52 at a reduced operating voltage of 2 V, along with improved endurance characteristics. These results demonstrate a viable strategy for engineering high‐κ ferroelectric capacitors with low‐voltage operation, making them promising candidates for advanced DRAM and nonvolatile memory applications.

## Graphical abstract

The Hf_x_Zr_1‐x_O_2_‐based ferroelectric/antiferroelectric bilayer capacitor exhibits morphotropic‐phase‐boundary behavior with a high dielectric constant (∼52) at 2 V. Phase engineering stabilizes o/t‐phase coexistence and suppresses m‐phase formation, enabling capacitance enhancement and self‐optimization under cycling for scalable low‐voltage, high‐κ memory integration.

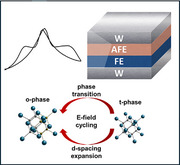

## 1 Introduction

Dynamic random‐access memory (DRAM) technology has advanced through aggressive cell‐size scaling to achieve higher integration densities and faster operation. However, as the physical cell size continues to shrink, the capacitor area becomes increasingly limited, making it challenging to secure sufficient capacitance required for maintaining reliable signal‐to‐noise ratio and stable sensing margin. Adequate capacitance remains a critical factor for ensuring DRAM performance, especially as scaling approaches sub‐10 nm nodes [1, 2].

To address the diminishing capacitor area, high‐κ dielectrics have been extensively investigated as alternatives to conventional SiO_2_/Al_2_O_3_‐based materials. Oxides such as SrTiO_3_, BaSrTiO_3_, TiO_2_, and Al‐doped TiO_2_ (κ ≥ 100) offer significantly higher permittivity [3, 4, 5]. However, their relatively narrow bandgaps (∼3.2–3.3 eV) often result in excessive leakage currents, while interface instability and poor crystallinity with TiN electrodes further limit their integration into standard CMOS processes.

In contrast, hafnium‐zirconium oxide (Hf_x_Zr_1−x_O_2_, HZO) has emerged as a highly promising next‐generation DRAM capacitor dielectric due to its wider bandgap (∼5.5 eV), excellent CMOS compatibility, and lower leakage current [6, 7]. Alloying HfO_2_ with ZrO_2_ not only reduces the crystallization temperature but also enables the stabilization of ferroelectric phases. Particularly, compositions near the morphotropic phase boundary (MPB), where o‐ and t‐phases coexist, exhibit maximized dielectric constant, minimized equivalent oxide thickness (EOT), and enhanced endurance [8, 9, 10]. Consequently, substantial research efforts have focused on optimizing HZO structural and processing parameters, including composition, film thickness, annealing conditions, electrode materials, and electric field cycling [11, 12, 13, 14, 15, 16, 17].

In this work, we explore the use of bilayer HZO structures, combining antiferroelectric (AFE) and ferroelectric (FE) layers, as a strategy to promote stable MPB formation over a wider processing window. Through systematic variation of composition, individual layer thicknesses, annealing conditions, electrode selection, and field‐cycling protocols, we investigate phase evolution, dielectric response, and electrical characteristics. Our approach aims to simultaneously achieve ultrathin EOT, suppressed leakage current, and enhanced dielectric constant, thereby providing design guidelines for integrating bilayer‐engineered HZO dielectrics into advanced DRAM capacitors [18].

## 2 Results and Discussion

### 2.1 Thickness Ratio Effects on Dielectric and Polarization Behavior

**FIGURE 1:**
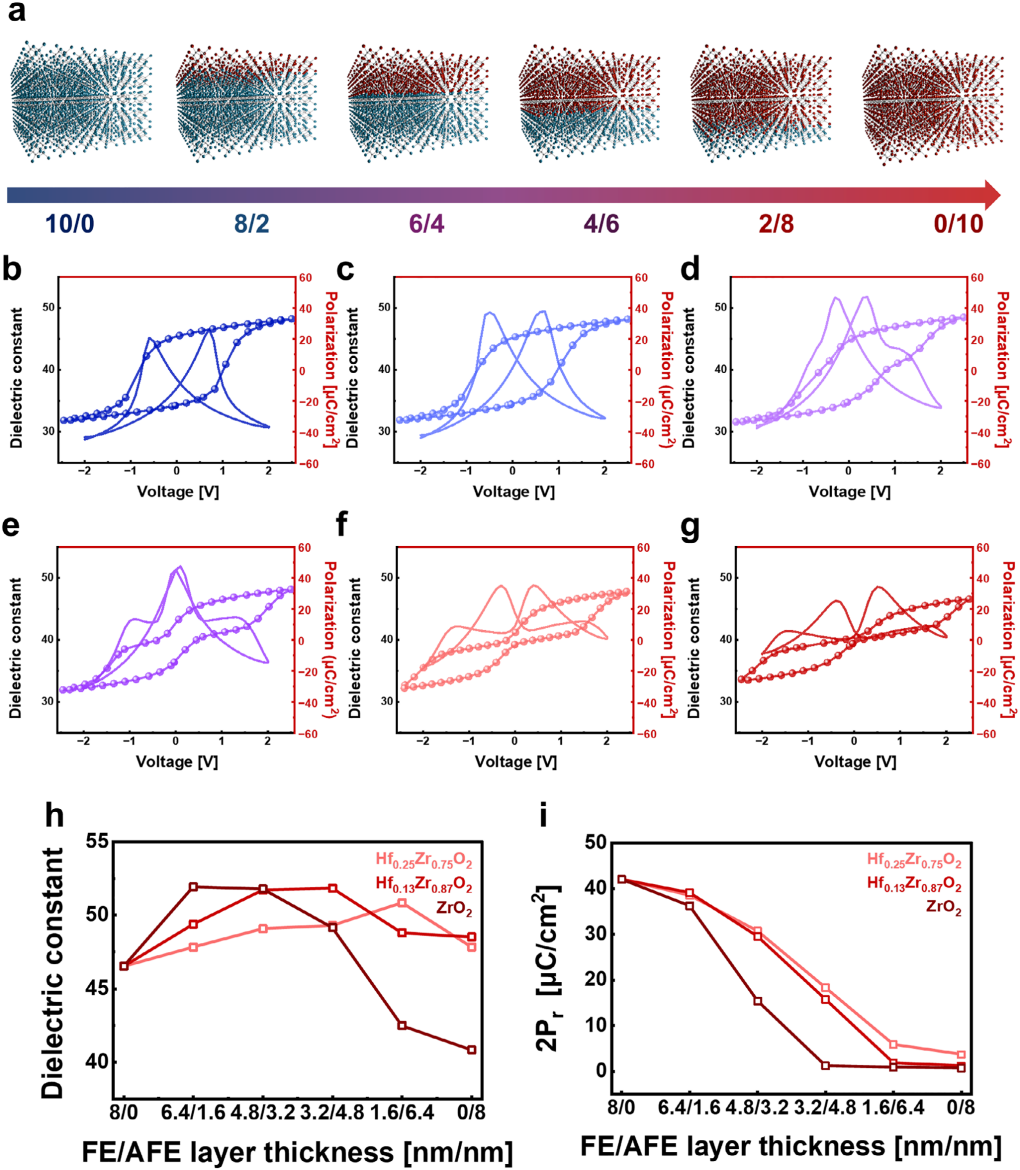
(a) Schematic illustration of FE/AFE bilayer capacitors with varying thickness ratios. Blue and red represent FE and AFE layers, respectively. (b–g) Dual‐sweep dielectric constant–voltage (κ–V) characteristics measured at 2 V and polarization–voltage (P–V) hysteresis loops measured at 2.5 V for FE/AFE bilayers with different thickness configurations at Hf_0.13_Zr_0.87_O_2_ composition. (h) Dielectric constant and (i) remnant polarization (2P_r_) of FE/AFE bilayers with different AFE compositions (Hf_0.25_Zr_0.75_O_2_, Hf_0.13_Zr_0.87_O_2_, and ZrO_2_).

Figure 1a illustrates the FE/AFE bilayer capacitor based on Hf_x_Zr_1−x_O_2_ (HZO). The bottom layer adopts a ferroelectric composition of Hf_0.5_Zr_0.5_O_2_, while the top layer consists of AFE‐dominant compositions of Hf_0.25_Zr_0.75_O_2_, Hf_0.13_Zr_0.87_O_2_, and ZrO_2_. As shown in Figure 1b–g, the 8/0 and 6.4/1.6 bilayers show typical butterfly‐shaped κ–V curves characteristic of the orthorhombic (o‐) phase, reflecting ferroelectric polarization switching. With increasing thickness of the AFE top layer, the 4.8/3.2 and 3.2/4.8 bilayers exhibit enhanced dielectric response and pinched κ–V profiles, indicating the coexistence of o‐ and t‐phases near the morphotropic phase boundary (MPB). When the Hf:Zr ratio approaches 60%–70%, the dielectric constant reaches its maximum. In contrast, the 1.6/6.4 and 0/8 bilayers display a distinct double‐hump feature originating from field‐induced antiferroelectric transitions in ZrO_2_ [19, 20].

These phase transitions are further supported by P–V hysteresis loops. The FE‐dominant structures such as 8/0 and 6.4/1.6 configurations exhibit well‐saturated loops with large remanent polarization, whereas the MPB bilayers show reduced coercive field (E_c_) and slanted loops, indicating partial o‐phase switching. AFE‐dominant structures such as 1.6/6.4, 0/8 configurations show negligible remanent polarization, consistent with non‐volatile AFE behavior. As summarized in Figure 1h,i, the dielectric constant peaks at an effective Hf:Zr ratio of near 3:7, while 2Pr decreases monotonically with increasing Zr content. These trends highlight that dielectric response is primarily governed by composition rather than specific AFE phase selection.

### 2.2 Phase Composition and Dielectric Enhancement near MPB

**FIGURE 2:**
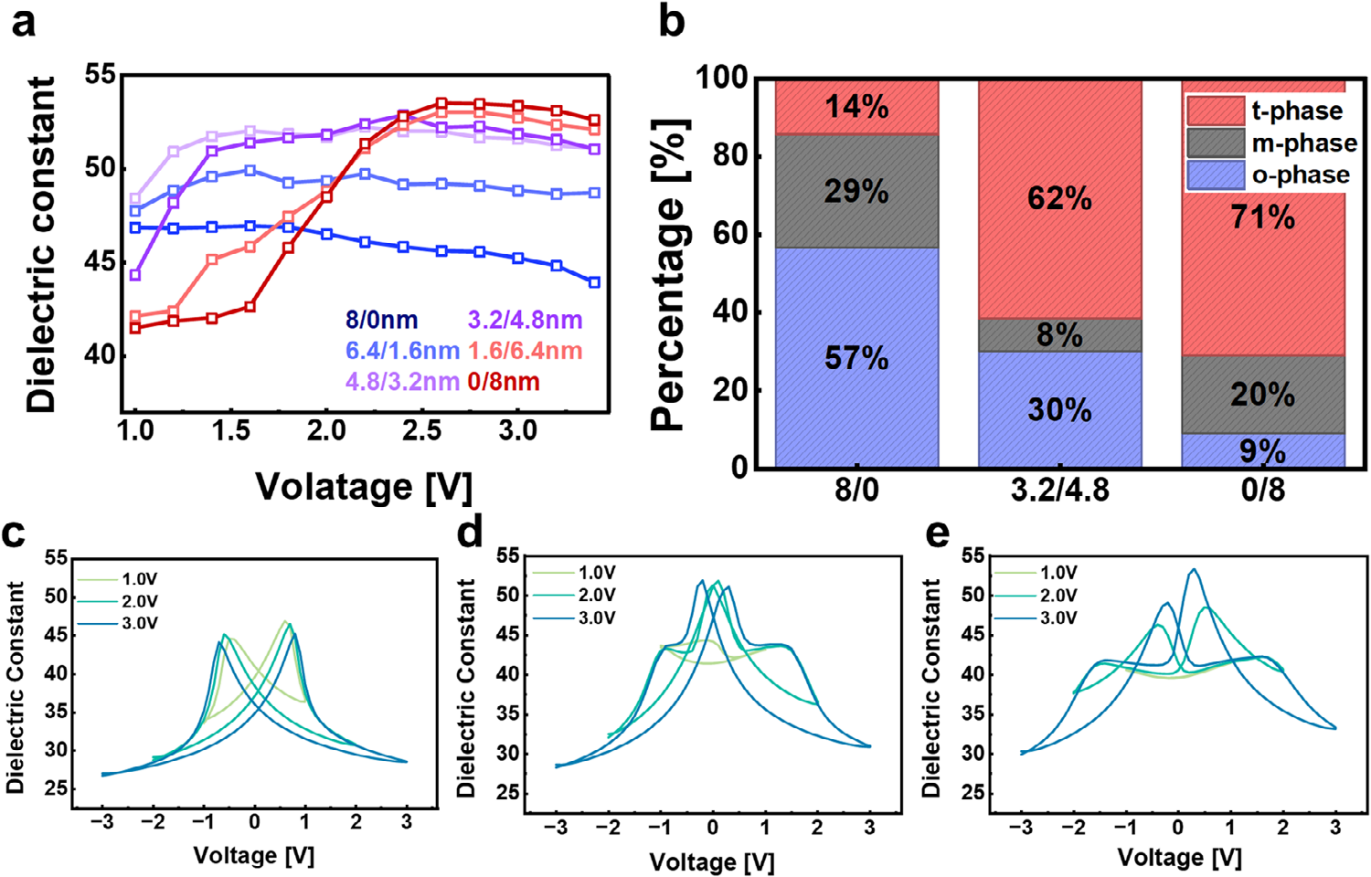
(a) Maximum dielectric constant values of Hf_0.13_Zr_0.87_O_2_ ‐based single‐layer and bilayer capacitors measured under DC bias voltages from 1.0 to 3.4 V with 0.2 V intervals. (b) Phase fractions of o‐, t‐, and m‐phases extracted from XRD analysis for representative structures (8.0,3.2/4.8, and 0/8). (c–e) C–V curve of (c) 8 nm FE‐HZO, (d) 3.2/4.8 nm FE/AFE bilayer, and (e) 8 nm AFE‐HZO films at the same composition.

Figure 2a presents the bias‐dependent dielectric behavior of Hf_0.13_Zr_0.87_O_2_ ‐based single‐layer and bilayer capacitors. The maximum dielectric constant at each DC bias was extracted from the corresponding C–V curve as the highest capacitance value within the applied operating voltage range. As the AFE layer becomes thicker, the voltage at which the maximum capacitance appears gradually shifts to higher bias. FE‐dominant structures 8/0 and 6.4/1.6 show a monotonic decrease in dielectric constant after an initial rise, while MPB bilayers 4.8/3.2 and 3.2/4.8 exhibit a pronounced increase in κ at low voltages followed by a gradual reduction at higher bias. In contrast, AFE‐dominant devices 1.6/6.4 and 0/8 show a continuous increase in κ with increasing bias, corresponding to field‐induced antiferroelectric transitions.

The detailed C–V characteristics shown in Figure 2c–e reveal these distinctions more clearly. The 6.4/1.6 devices, dominated by a thick ferroelectric bottom layer, behave similarly to the FE single layer but exhibit slightly higher capacitance due to the influence of the thin AFE top layer. Their butterfly‐shaped κ–V profiles show capacitance rising steeply from low fields and saturating near 1 V, indicating rapid o‐phase polarization switching and a fast low‐voltage response.

When the ratio between FE and AFE layers approaches balance, as in the 4.8/3.2 and 3.2/4.8 bilayers, the devices exhibit strongly voltage‐dependent dielectric responses. At 1 V, the κ–V curves deviate from typical AFE‐like profiles; at 2 V, both o‐ and t‐phase dielectric characteristics appear, forming a pinched κ–V curve with merged peaks near 0 V, typical of the MPB region. At 3 V, the curves evolve into slightly pinched butterfly‐like shapes. These observations indicate that MPB capacitors show distinct voltage‐dependent dielectric behavior, emphasizing the need to optimize operating voltage for stable performance.

As the AFE layer becomes thicker in 1.6/6.4 and 0/8 devices, the capacitance response is negligible at low bias but develops a double‐humped AFE‐type profile around 2 V. At 3 V, the dielectric constant further increases, and the curve begins to resemble that of the MPB bilayer; however, breakdown occurs before a complete MPB‐like transition can take place due to excessive electric‐field stress. As a result, the bilayer structures achieve high dielectric constants at operating voltages as low as 1.8 V, whereas single‐layer capacitors require more than 2.5 V to reach comparable levels.

To clarify the structural origin of these dielectric differences, GI‐XRD analysis was performed to examine phase evolution as a function of the FE/AFE thickness ratio. As shown in Figure 2b, the GI‐XRD patterns of representative 8/0, 3.2/4.8, and 0/8 samples corroborate the electrical observations and confirm that the enhanced dielectric response is closely linked to phase composition [21, 22]. The o‐phase fraction decreases as the FE layer becomes thinner, while the t‐phase fraction increases with the growth of the AFE layer. Consequently, o‐phase dominates in FE‐type structures, t‐phase in AFE‐type structures, and bilayers exhibit slightly t‐phase‐dominant coexistence of both, reflecting compositional trends in phase stability rather than a strictly phase‐separated structure. Notably, the 3.2/4.8 MPB bilayer suppresses m‐phase formation below approximately 8%, owing to competitive nucleation of o‐ and t‐phases at balanced FE/AFE interfaces [23, 24]. This structural stabilization near the MPB composition provides a clear physical basis for the observed high dielectric constant and low‐voltage operation in the bilayer capacitors.

### 2.3 Voltage Dependent Switching of MPB Bilayer Capacitor

**FIGURE 3:**
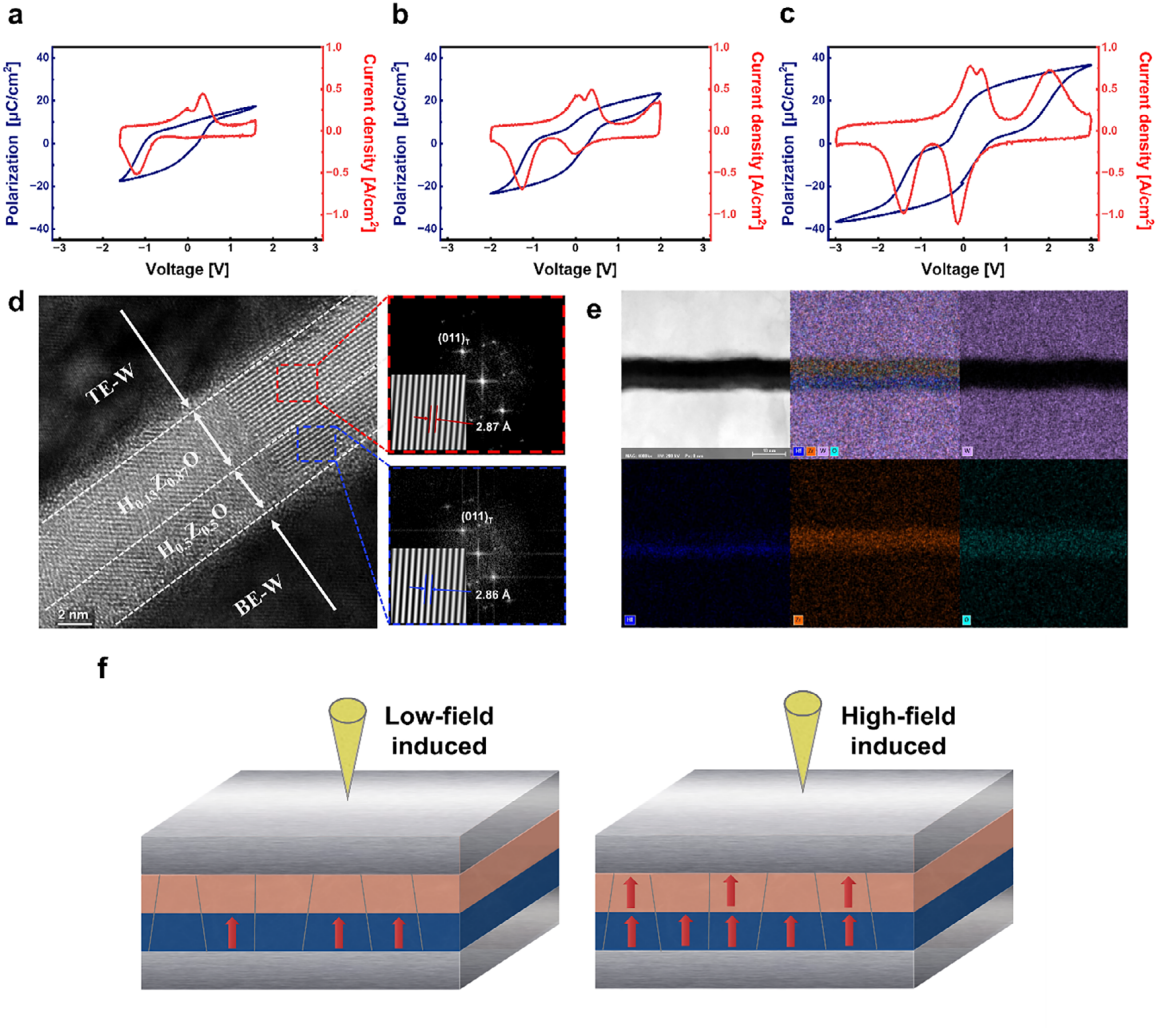
(a–c) P–V and J–V characteristics of the 3.2/4.8 nm Hf_0.13_Zr_0.87_O_2_ bilayer capacitor measured at (a) 1.6, (b) 2.0, and (c) 3.0 V. (d) Cross‐sectional STEM image with FFT patterns showing distinct crystalline layers. (e) EDS elemental maps confirming sharp Hf/Zr interfaces. (f) Schematics of low‐ and high‐field‐induced polarization switching in the FE/AFE bilayer.

As shown in Figure 3a, at a bias of 1.6 V the polarization switching is highly asymmetric: polarization along the positive direction is largely suppressed, while the negative branch shows a clear switching response. A small current peak near 0 V, attributed to the AFE component, is observed, whereas the o‐phase–related peak that typically appears near 2 V is absent. Figure 3b illustrates the response at 2.0 V. In this regime, the negative branch exhibits a nearly complete polarization reversal, whereas the positive branch remains incomplete. A new current peak emerges near 2 V, suggesting the onset of o‐phase activation. This corresponds to the voltage range where the device attains its maximum dielectric constant, indicating that the upper AFE layer begins to participate in switching. At a higher bias of 3.0 V, as shown in Figure 3c, a fully developed P–V loop is obtained, displaying a symmetric hysteresis and higher current density. This behavior reflects collective domain activation in both the FE bottom layer and the AFE‐dominant top layer, resulting in complete polarization switching under high field.

Structural analysis supports this interpretation. As shown in Figure 3d, the cross‐sectional STEM image reveals that the HZO bilayer crystallizes uniformly across the stack, without a distinct phase boundary between the top and bottom layers. This observation is consistent with previous crystallization studies of HZO bilayers and confirms that the two layers share similar crystallographic phases rather than forming a well‐defined O/T bilayer [25]. Therefore, the distinct polarization behaviors observed in the bilayer device are not attributed to differences in crystal phase but instead arise from the compositional asymmetry between the top and bottom regions, which leads to different effective switching fields and polarization responses under an applied electric field. Rather, as confirmed by Figure 3e, the elemental‐mapping results show a compositional gradient in the Hf:Zr ratio across the film, implying that the compositional variation governs the observed field‐dependent asymmetry.

The schematic diagrams in Figure 3f summarize the proposed mechanism. At low voltage, only the FE bottom layer contributes to switching, while the AFE top layer remains inactive, producing an asymmetric curve with a suppressed positive‐field response. Although interfacial diffusion inevitably results in a gradual compositional profile rather than a sharp interface, the difference in effective switching thresholds is still preserved, enabling preferential switching in the bottom region. When the voltage is increased, more domains in the FE layer are aligned, and the AFE layer becomes responsive, yielding a symmetric loop with higher current density. This field‐driven activation of the AFE component explains the transition from asymmetric to symmetric polarization behavior in the FE/AFE bilayer capacitor.

### 2.4 Endurance Characteristics and Capacitance Wake‐Up Effect

**FIGURE 4:**
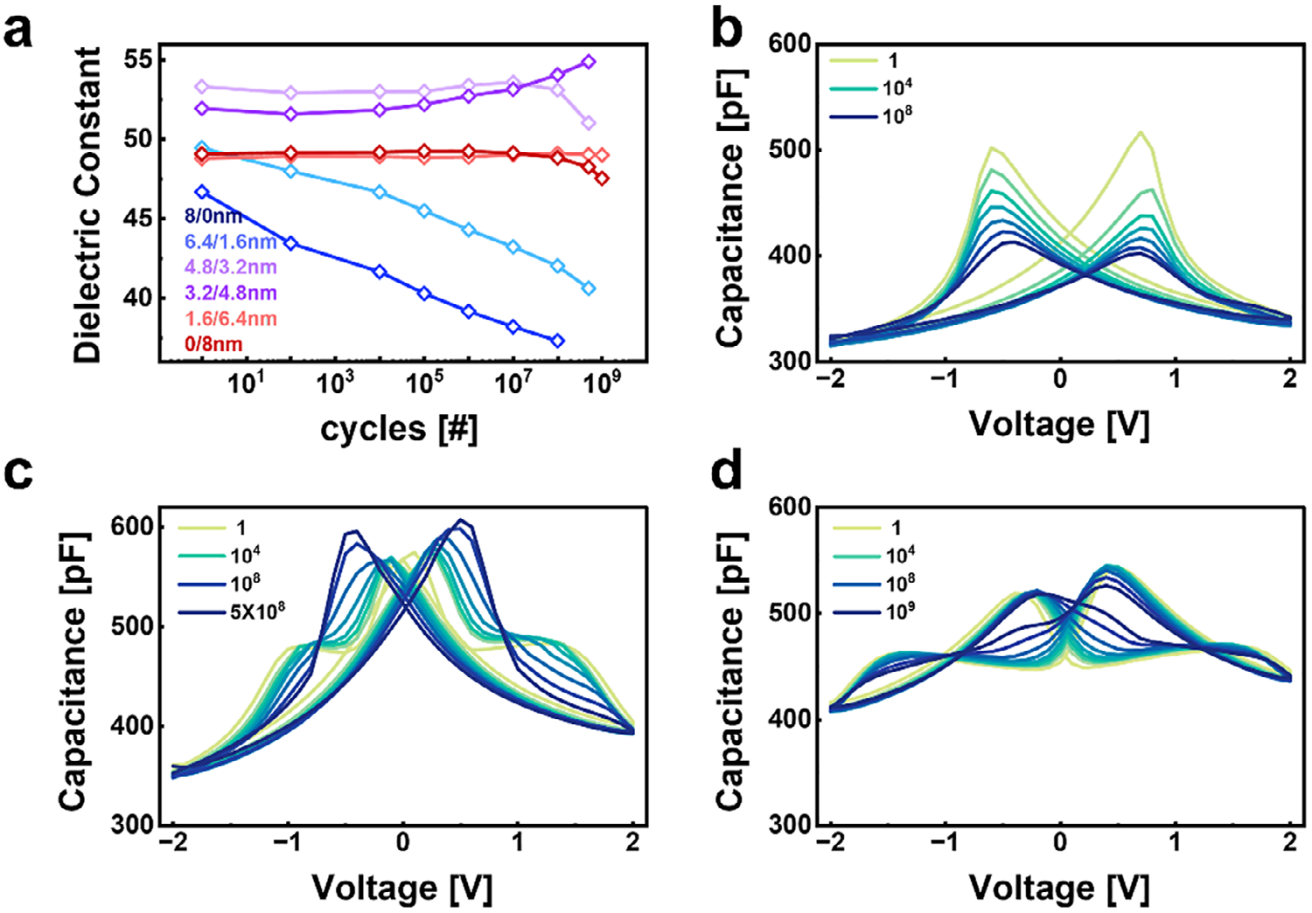
(a) Endurance characteristics of Hf_0.13_Zr_0.87_O_2_‐based single‐layer and bilayer capacitors measured up to 10^9^ cycles. (b–d) Evolution of C–V curves during cycling for (b) FE‐HZO (8/0), (c) AFE‐HZO (0/8), and (d) FE/AFE bilayer (3.2/4.8 nm) capacitors.

Figure 4a shows that the operational stability of Hf_0.13_Zr_0.87_O_2_ capacitors strongly depends on the thickness ratio between the ferroelectric and antiferroelectric layers. The endurance characteristics were evaluated using a bipolar triangular cycling waveform with a fixed amplitude of ±2 V, as schematically illustrated in Figure S7. Ferroelectric‐dominant structures such as 8/0 and 6.4/1.6 initially exhibit high dielectric constants but gradually degrade during cycling, showing fatigue similar to the previously reported reduction of remanent polarization in ferroelectric films. As shown in Figure 4b, the butterfly‐shaped ferroelectric response continuously shrinks as the initial orthorhombic phase transforms into the nonpolar monoclinic phase under repeated electric‐field stress. Although Hf‐rich single layers provide excellent low‐voltage operation, they show limited long‐term reliability [26, 27, 28]. The reduction in dielectric constant and the appearance of asymmetric hysteresis loops result from a decrease in the number of switchable orthorhombic domains caused by domain pinning and the growth of inactive interfacial regions.

In contrast, antiferroelectric‐dominant structures such as 1.6/6.4 and 0/8 show almost no capacitance degradation even after 10^9^ cycles, as shown in Figure 4c. Zr‐rich films rely on field‐induced transitions between nonpolar and polar states, making them less vulnerable to irreversible fatigue caused by domain collapse. In the early stages of cycling, the characteristic double‐humped capacitance‐voltage curves remain clearly visible, but as cycling continues, asymmetry and imbalance between the two peaks gradually increase, indicating a progressive loss of symmetry. Although the overall capacitance remains stable, the double‐humped profile becomes increasingly distorted. This behavior agrees with earlier reports showing that antiferroelectric HZO maintains high cycling endurance despite negligible remanent polarization, while its peak symmetry can degrade under prolonged operation.

The most distinct behavior appears in the morphotropic phase boundary bilayers, particularly the 3.2/4.8 configuration. As shown in Figure 4d, the bilayer maintains increasing capacitance characteristics during cycling, without the high‐field‐induced degradation observed in ferroelectric single layers [29, 30, 31]. Within an appropriate operating voltage range, the electric field balances the contributions of both layers, preventing excessive polarization rotation and domain pinning. As a result, the bilayer undergoes gradual ordering of dipoles and interfacial domain alignment, leading to self‐optimization of the dielectric response. This demonstrates that the morphotropic phase boundary configuration provides a naturally stable operating field, ensuring consistent dielectric behavior and improved endurance during prolonged cycling.

In the 4.8/3.2 configuration, an excessive accumulation of the orthorhombic phase after around 10^7^ leads to fatigue, whereas higher Zr content mitigates this effect by stabilizing the tetragonal phase and suppressing the formation of inactive regions. Previous studies have reported a decrease in tetragonal peak intensity and a corresponding increase in orthorhombic peaks in HZO near the morphotropic boundary during electric‐field cycling, which confirms phase redistribution. In this study, compositions within the boundary region, including Hf_0.25_Zr_0.75_O_2_ and Hf_0.13_Zr_0.87_O_2_ bilayers with 3.2/4.8 thickness ratios as well as Zr‐rich 4.8/3.2 structures, exhibit the same transition from the tetragonal to the orthorhombic phase, resulting in higher capacitance compared to the initial state. The complementary composition of the bilayer provides an energetically favorable interface that facilitates field‐driven phase reorganization.

Therefore, morphotropic phase boundary bilayers show a clear tendency toward self‐optimization, where device performance improves during cycling. This demonstrates that bilayer design is not only effective in suppressing degradation but also provides a practical pathway to achieving both low‐voltage operation and long‐term endurance.

### 2.5 Phase Transition and Lattice Expansion Under Field Cycling

**FIGURE 5:**
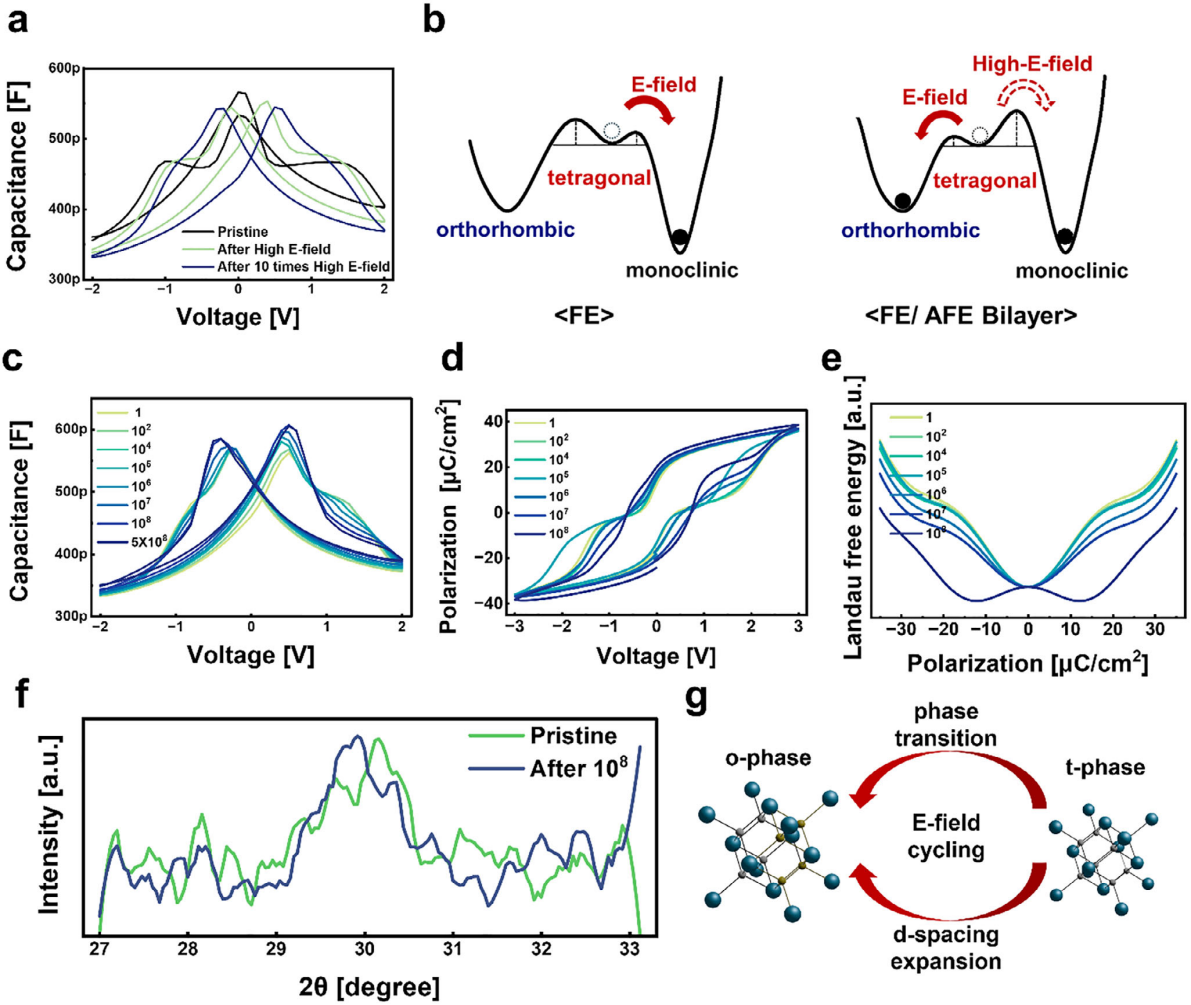
(a) C–V characteristics of the 3.2/4.8 nm Hf_0.13_Zr_0.87_O_2_ bilayer capacitor in the pristine state and after high‐field stressing at 3 V. (b) Calculated Gibbs free‐energy profiles of single‐layer FE and FE/AFE bilayer structures under increasing electric field. (c–e) Evolution of (c) C–V, (d) P–V, and (e) free‐energy curves during endurance cycling up to 10^8^ cycles. (f) XRD spectra of the bilayer before and after 10^8^ cycles showing peak shifts associated with the phase transition. (g) Schematic illustration of field‐induced phase transformation accompanied by d‐spacing expansion.

As shown in Figure 5a, although the pristine state exhibits a morphotropic‐boundary‐like capacitance profile, the application of a strong electric field above 3 V triggers a phase transition toward the orthorhombic phase. Continuous stressing at 3 V induces successive transformations, indicating that the bilayer is highly sensitive to field strength. At moderate fields (around 2 V), the transition is dominated by a tetragonal‐to‐orthorhombic conversion; however, when the applied field exceeds 3 V, additional transition pathways become accessible. At appropriate operating voltages, repeated cycling enhances the dielectric response through gradual phase reorganization; however, under excessively high electric fields, the capacitance undergoes a sustained decrease due to irreversible structural rearrangement and phase stabilization.

This behavior originates from a fundamental modification of the Gibbs free‐energy landscape, as illustrated in Figure 5b [23]. In single‐layer Hf_0.5_Zr_0.5_O_2_, the energy barrier between the tetragonal and monoclinic phases is relatively low, making the tetragonal phase prone to transform into the monoclinic phase under sustained electric fields. This transition leads to the formation of interfacial dead layers and results in a gradual reduction in capacitance and remanent polarization. In contrast, the asymmetric composition of the bilayer introduces competition between the orthorhombic and tetragonal phases, which effectively lowers the energy barrier for the tetragonal‐to‐orthorhombic transition. As a result, when an appropriate electric field is applied, the tetragonal phase predominantly transforms into the orthorhombic phase, leading to irreversible phase reorganization and enhanced dielectric response. At high‐field operation, the capacitance response exhibits a butterfly‐shaped characteristic accompanied by a reduction in capacitance, indicating a change in the underlying switching behavior compared to the low‐field regime.

When the applied voltage exceeds 3 V, the electric field becomes sufficiently strong to access not only the barrier associated with the tetragonal‐to‐orthorhombic (T→O) transition, which is generally considered dominant, but also higher‐energy pathways. In this high‐field regime, the involvement of an additional tetragonal‐to‐monoclinic (T→M) transition cannot be excluded, suggesting that the electrical response may not be solely governed by the T→O transition under such conditions.

As shown in Figure 5c, subsequent biasing at 2 V restores the orthorhombic phase and recovers the capacitance to its previous level, demonstrating that the bilayer retains an inherent self‐recovery capability when operated within the appropriate voltage range. This can be interpreted as evidence suggesting that, while the T→O transition dominates in the intermediate electric‐field regime, the involvement of a T→M transition cannot be excluded under strong electric‐field conditions. As endurance cycling proceeds, the evolution of the electrical and structural characteristics becomes evident. Figure 5d shows that the polarization–voltage response gradually recovers and becomes more symmetric with repeated cycling, reflecting progressive stabilization of the orthorhombic domains and partial suppression of the antiferroelectric contribution.

Figure 5e shows the evolution of the Landau‐calculated free‐energy profiles during endurance cycling. In the early stage, the energy surface exhibits a relatively flat profile near zero bias, reflecting the coexistence of multiple metastable states at the morphotropic boundary [32, 33]. With continued cycling, the double‐well potential characteristic of the orthorhombic ferroelectric phase gradually emerges, while the tetragonal well becomes shallower and eventually merges into a single, deeper minimum. This transition indicates progressive stabilization of the orthorhombic polarization state and a reduction of the overall energy barrier, which facilitates polarization switching and explains the enhanced dielectric response observed electrically.

Consistent with this theoretical prediction, Figure 5f provides direct structural evidence of such evolution. Micro‐GI‐XRD measurements reveal that after 10^8^ cycles, the diffraction peaks of the 3.2/4.8 nm bilayer shift globally toward lower 2*θ* angles, indicating an overall expansion of the lattice spacing according to Bragg's law [34]. In addition, the main peak, which was initially located at higher 2*θ* values and dominated by the tetragonal phase, moves toward the center of the profile after cycling, signifying an increased contribution from the orthorhombic phase. These observations demonstrate that both the overall lattice expansion and the phase transformation from tetragonal to orthorhombic occur simultaneously during endurance cycling. The enlarged d‐spacing reduces the restoring force for ionic displacement, thereby enhancing local polarizability and thereby the effective dielectric response, which explains the continued capacitance increase even though the orthorhombic phase intrinsically possesses a lower permittivity than the tetragonal phase.

Figure 5g schematically summarizes this process. Repeated electric‐field cycling drives the conversion from tetragonal to orthorhombic phases while simultaneously expanding the lattice spacing. This dual mechanism—field‐induced phase transformation combined with d‐spacing expansion—accounts for both the progressive capacitance enhancement and the self‐recovery behavior observed when the device operates within its optimal voltage range.

As demonstrated in the previous results, defining an appropriate operating‐voltage range is critical for optimizing the performance of morphotropic‐phase‐boundary (MPB) capacitors. While a high dielectric constant can be obtained under strong electric fields, such conditions lead to reduced dielectric responsiveness and accelerated endurance degradation. Therefore, operating the capacitor within the MPB voltage window is essential to maintain both high dielectric efficiency and long‐term stability.

**FIGURE 6:**
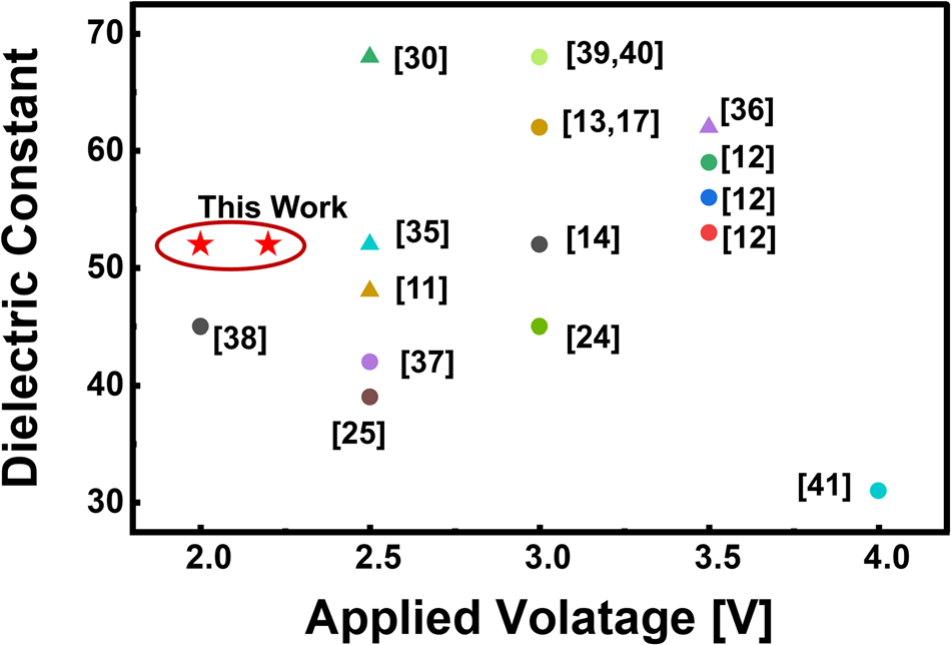
Benchmark of dielectric constant vs. sweep voltage for Hf‐based capacitors. Triangles represent devices using annealing techniques, while circles denote those improved by compositional engineering [35, 36, 37, 38, 39, 40, 41].

Figure 6 benchmarks the dielectric performance of the proposed FE/AFE bilayer capacitor against previously reported HZO‐based systems. Compared with single‐layer capacitors that typically require operation above 3 V, the bilayer structure achieves nearly the same dielectric constant at only 2 V. This demonstrates that the bilayer design effectively reduces the operating field while preserving a high dielectric response, providing a practical pathway toward low‐voltage and endurance‐stable operation. Furthermore, integrating this bilayer architecture with advanced annealing techniques, such as laser or rapid thermal annealing, could further lower the operating voltage while maintaining a high dielectric constant. Such combined approaches hold significant potential for realizing ultralow‐voltage, high‐κ capacitors optimized for next‐generation DRAM and energy‐efficient logic devices.

## 3 Conclusion

This study provides a comprehensive understanding of morphotropic‐phase‐boundary (MPB) behavior in Hf_x_Zr_1−x_O_2_‐based ferroelectric/antiferroelectric bilayer capacitors. By engineering the bilayer structure, we demonstrated that the suppressed response of the top AFE layer can be effectively utilized to achieve a high dielectric constant of ∼52 at a low operating voltage of 2 V.

Without any additional annealing process, the compositional asymmetry in the bilayer stabilizes the coexistence of o‐ and t‐phases while suppressing the formation of the monoclinic phase. The effective operating‐voltage range of the MPB capacitor was identified, revealing that field‐induced t‐to‐o phase transitions and d‐spacing expansion occur during cycling, leading to self‐optimization with simultaneous enhancement and recovery of capacitance. Furthermore, the combination of a high dielectric response and a low operating voltage demonstrates the potential for low‐power and energy‐efficient operation, providing a practical route to voltage‐scalable ferroelectric devices.

Overall, this phase‐engineered bilayer framework offers a versatile platform that can be extended toward scalable multilayer capacitor stacks and embedded nonvolatile components for next‐generation DRAM, FeTFT, and logic technologies.

## 4 Experimental Section/Methods

The devices were fabricated on heavily doped p‐type (P^++^) silicon substrates. Prior to deposition, native oxides on the substrate surface were removed using a 6:1 buffered oxide etchant (BOE). Tungsten 55 nm was employed as the bottom electrode and deposited across the entire wafer by sputtering. Due to its high coefficient of thermal expansion, tungsten induces tensile stress in the HZO film, enhancing remanent polarization and promoting interfacial stability [42].

The HZO thin films were deposited by atomic layer deposition (ALD) at 250°C to a total thickness of 8 nm [43]. To control ferroelectric behavior, the Hf:Zr precursor cycle ratio was adjusted to 1:1 for ferroelectric layers and 1:3, 1:5, ZrO_2_ for antiferroelectric layers. The ALD process sequentially pulsed TEMAHf and TEMAZr precursors with H2O precursor to introduce Hf, Zr, and oxygen species. For top electrode formation, AZ 5214‐E photoresist was applied for lithographic patterning, followed by tungsten deposition under identical sputtering conditions as the bottom electrode (100 W, 2 mTorr, Ar flow rate: 20 sccm). The patterned electrodes were defined using a lift‐off process in acetone. Post‐deposition crystallization of the HZO films was performed using rapid thermal annealing at 600°C for 30 s in an N2 ambience. The fabricated MFM capacitors had an active area of 100 µm × 100 µm.

Electrical measurements were conducted at room temperature. P–V hysteresis loops and *J*–*V* curve were performed using a Radiant ferroelectric tester. Capacitance–voltage (*C*–*V*) characteristics, endurance, and leakage current were measured using a Keithley 4200 semiconductor parameter analyzer. *C*–*V* measurements were performed at 10 kHz using an AC excitation of 10 mV_rms_ with a bidirectional DC‐bias sweep from −V_max_ to + V_max_. Endurance measurements were performed with a pulse amplitude of 2 V. X‐ray diffraction (XRD) patterns were obtained using a PANalytical Empyrean diffractometer with Cu Kα radiation (λ = 1.5406 Å) operated at 40 kV and 30 mA.

## Funding

This work was supported by K‐CHIPS (Korea Collaborative & High‐tech Initiative for Prospective Semiconductor Research) (2410012306, 02305570, 25069‐15FC) funded by the Ministry of Trade, Industry & Energy (MOTIE, Korea). This work was supported by the National Research Foundation of Korea (NRF) grant funded by the Korea government (MSIT) (No. RS‐2023‐00260527).

## Conflicts of Interest

The authors declare no conflicts of interest.

## Supporting information




**Supporting File**: advs74328‐sup‐0001‐SuppMat.docx.

## Data Availability

The data that support the findings of this study are available in the supplementary material of this article.
