# Supplementary material for: Low‐Voltage and High‐k Properties of Bilayer HZO Capacitors at the Morphotropic Phase Boundary for Next‐Generation Memory Applications
Source: Adv Sci (Weinh). 2026 Feb 9;13(22):e19686. doi: 10.1002/advs.202519686 (PMC13088279; doi:10.1002/advs.202519686)
Supplement: Supplementary file 1 — Supporting File: advs74328‐sup‐0001‐SuppMat.docx. [file ADVS-13-e19686-s001.docx]

Supporting Information

**Low-Voltage and High-k Properties of Bilayer HZO Capacitors at the Morphotropic Phase Boundary for Next-Generation Memory Applications**

*Junseok Kim, Hyeonjung Park, Changwoo Han, Huiseong Shin, Myeongjae Choi and Changhwan Shin**

J. Kim, C. Han, H. Shin, C. Shin

School of Electrical Engineering

Korea University

Seoul 02841, Korea

E-mail: cshin@korea.ac.kr

H. Park

Department of Electrical and Computer Engineering

Sungkyunkwan University

Suwon 16419, Korea;

M. Choi

School of Semiconductor System Engineering

Sungkyunkwan University

Seoul 02841, Korea;


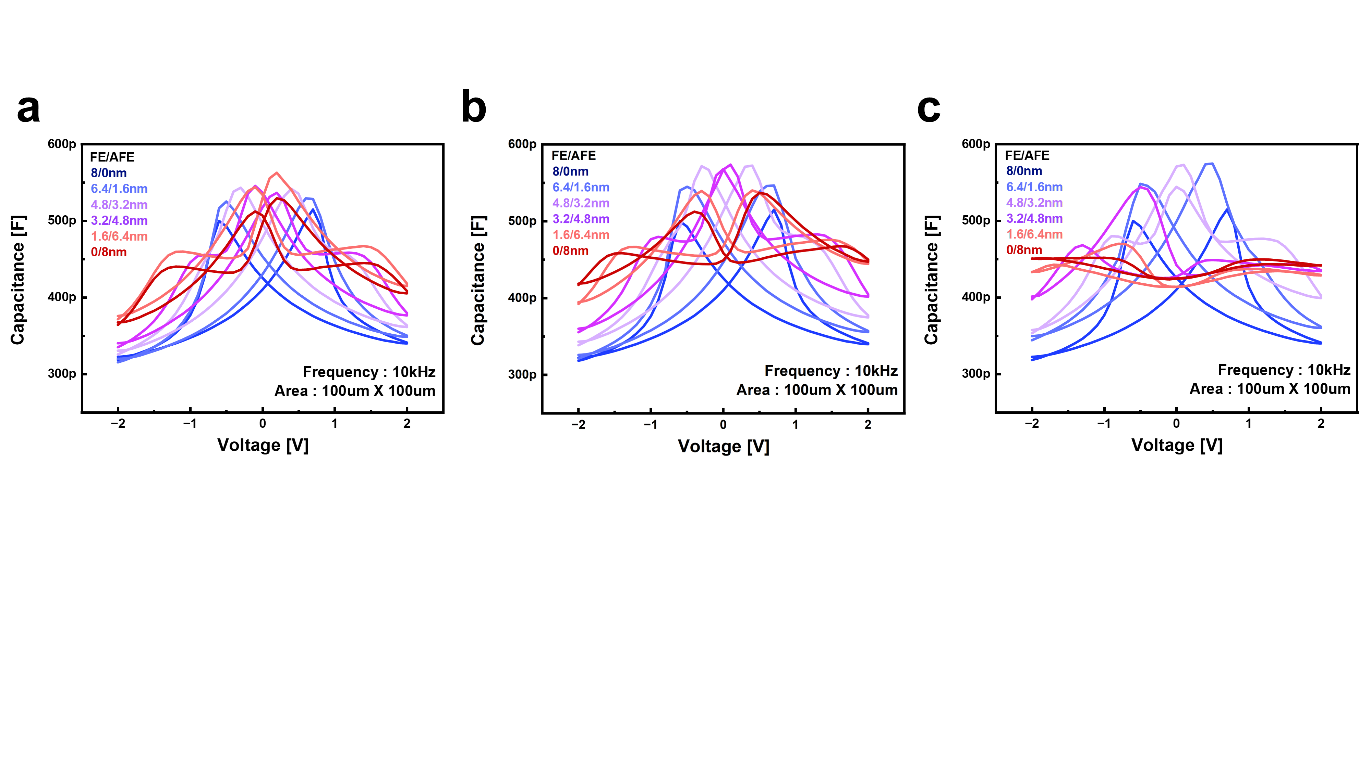


**Figure S1** **|** Capacitance–voltage (C–V) characteristics of FE/AFE bilayer capacitors with different thickness configurations measured at 10 kHz: (a) Hf_0.25_Zr_0.75_O_2_, (b) Hf_0.13_Zr_0.87_O_2_ and (c) ZrO_2_


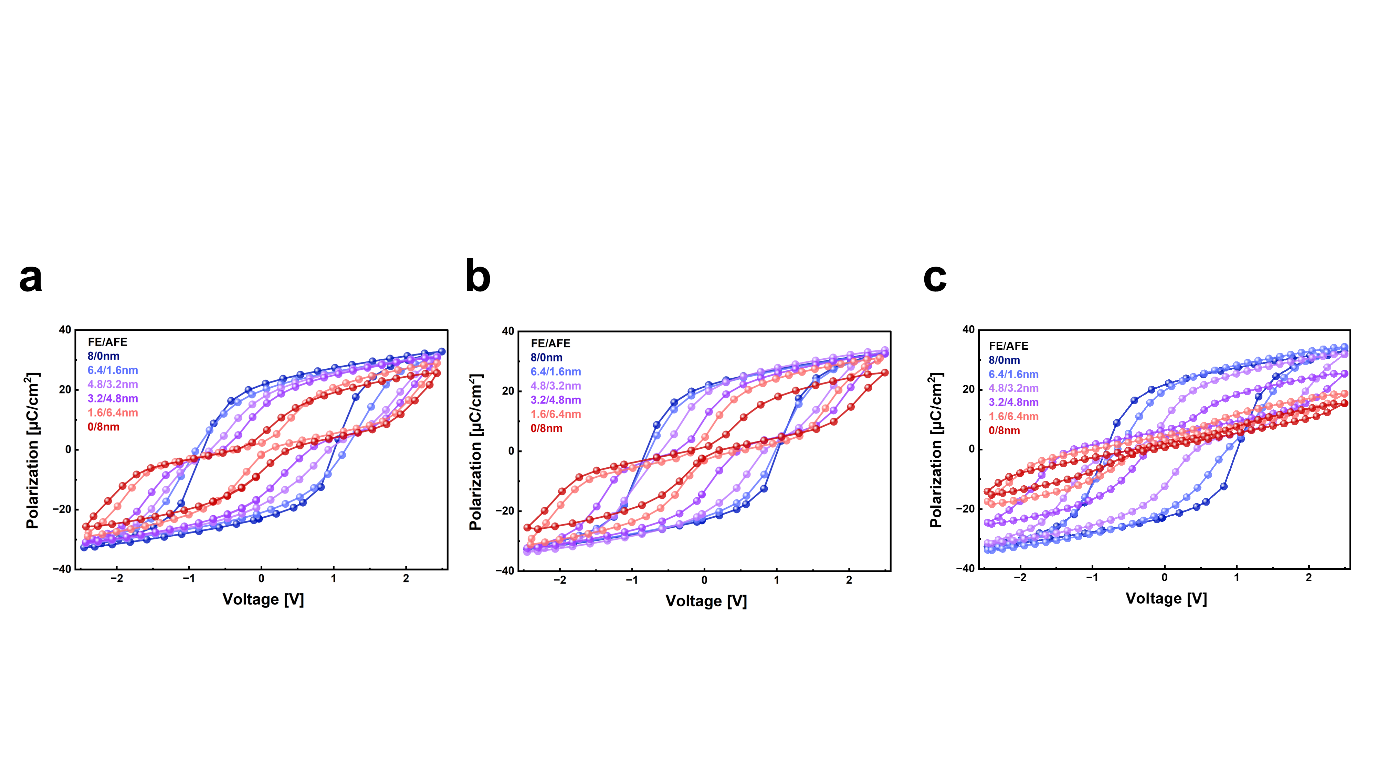


**Figure S2** **|** Polarization–voltage (P–V) hysteresis loops of FE/AFE bilayer capacitors with different thickness configurations: (a) Hf_0.25_Zr_0.75_O_2_, (b) Hf_0.13_Zr_0.87_O_2_ and (c) ZrO_2_


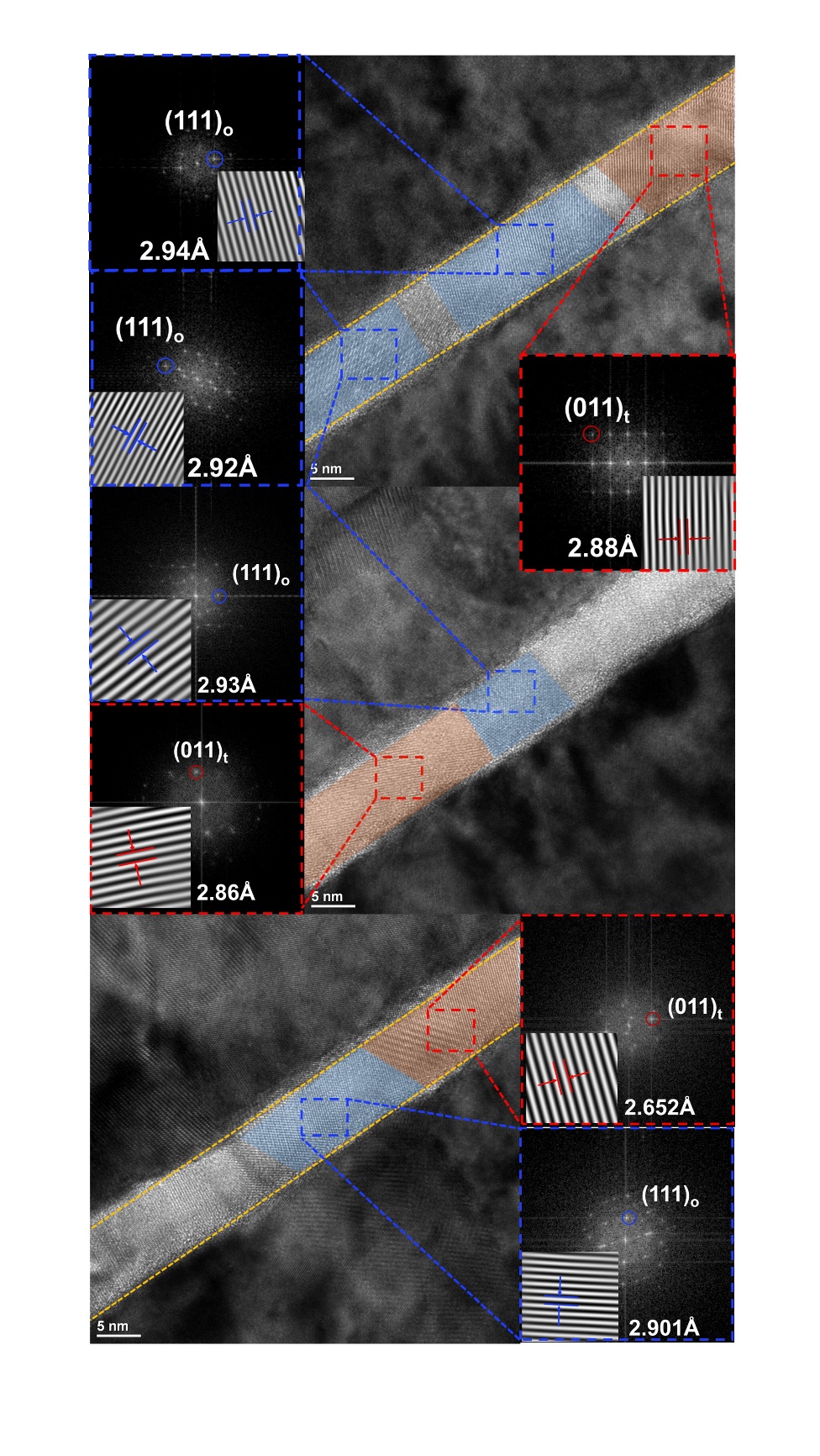


**Figure S3 |** S-TEM images acquired from three different regions of the 3.2/4.8 nm Hf_0.13_Zr_0.87_O_2_ bilayer capacitor. Each 5-nm field-of-view region shows the corresponding FFT pattern, identified crystal phase, polarization axis, and extracted d-spacing. Blue-marked FFT spots correspond to the orthorhombic (o-phase) with (111)_O_ reflections, while red-marked FFT spots correspond to the tetragonal (t-phase) with (011)t reflections.


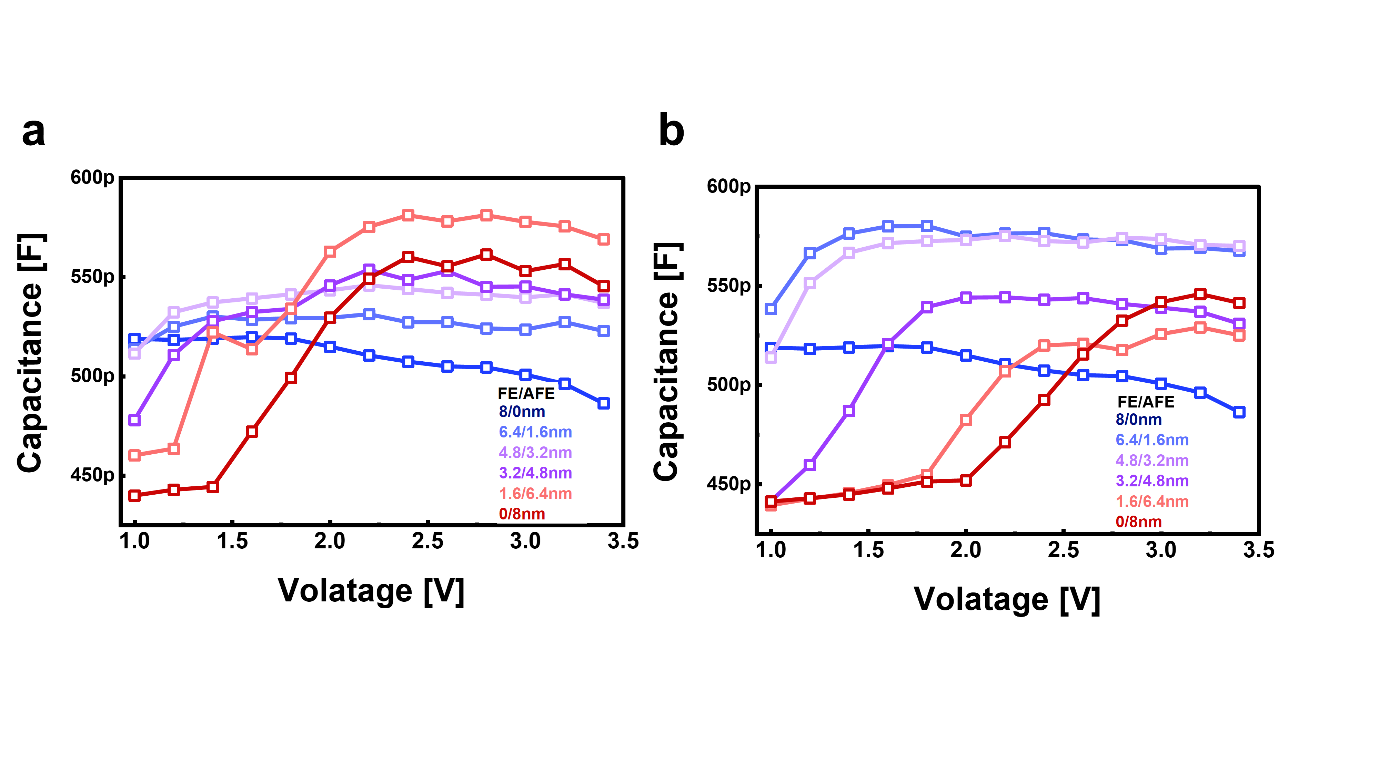


**Figure S4** **|** Maximum capacitance of FE/AFE bilayer capacitors with different thickness configurations as a function of applied voltage: (a) Hf_0.25_Zr_0.75_O_2_, (b) ZrO_2_


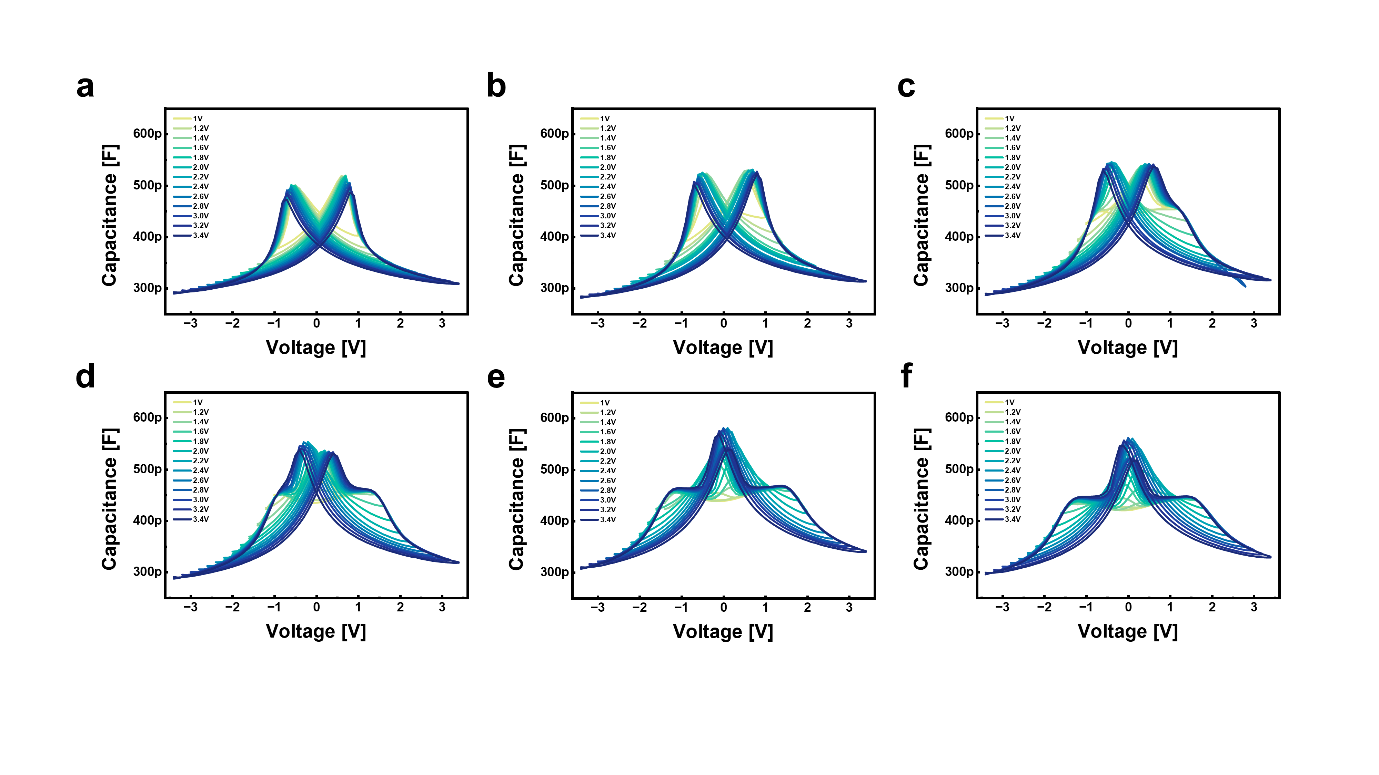

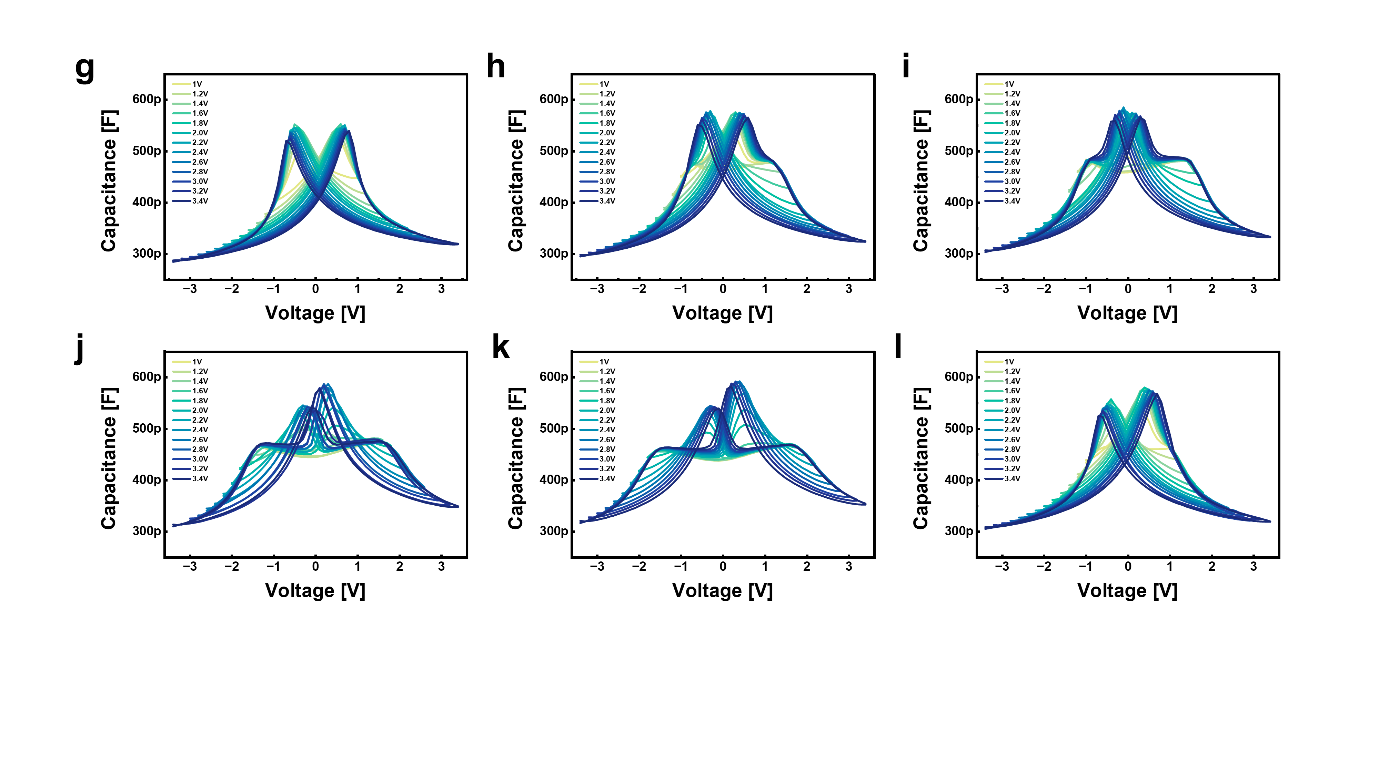


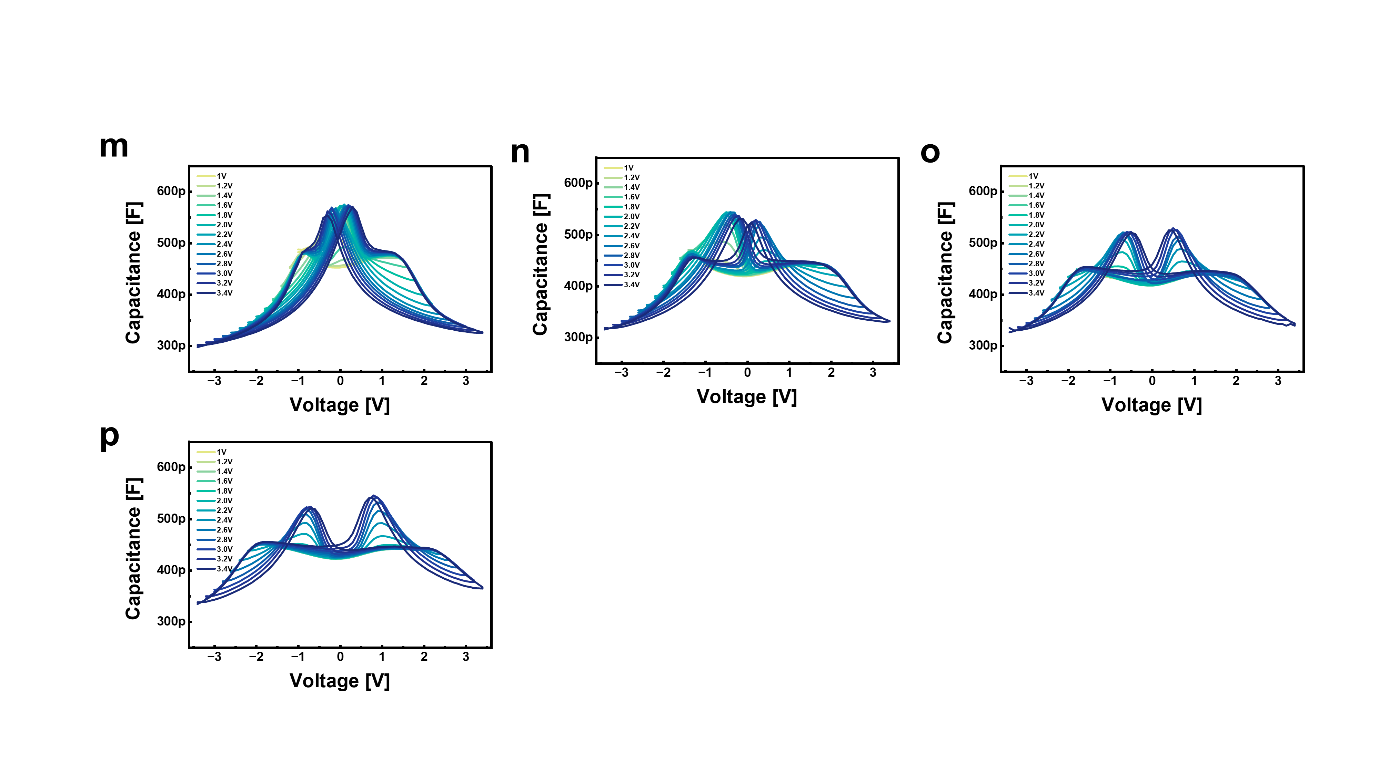


**Figure S5** **|** Capacitance–voltage (C–V) characteristics of FE/AFE bilayer capacitors with different thickness configurations: (a) 8/0, (b) 6.4/1.6, (c) 4.8/3.2, (d) 3.2/4.8, (e) 1.6/6.4, and (f) 0/8 nm at of Hf_0.25_Zr_0.75_O_2_ Composition, (g) 6.4/1.6, (h) 4.8/3.2, (i) 3.2/4.8, (j) 1.6/6.4, and (k) 0/8 nm at of Hf_0.13_Zr_0.87_O_2_ Composition, (l) 6.4/1.6, (m) 4.8/3.2, (n) 3.2/4.8, (o) 1.6/6.4, and (p) 0/8 nm at of ZrO_2_ Composition.


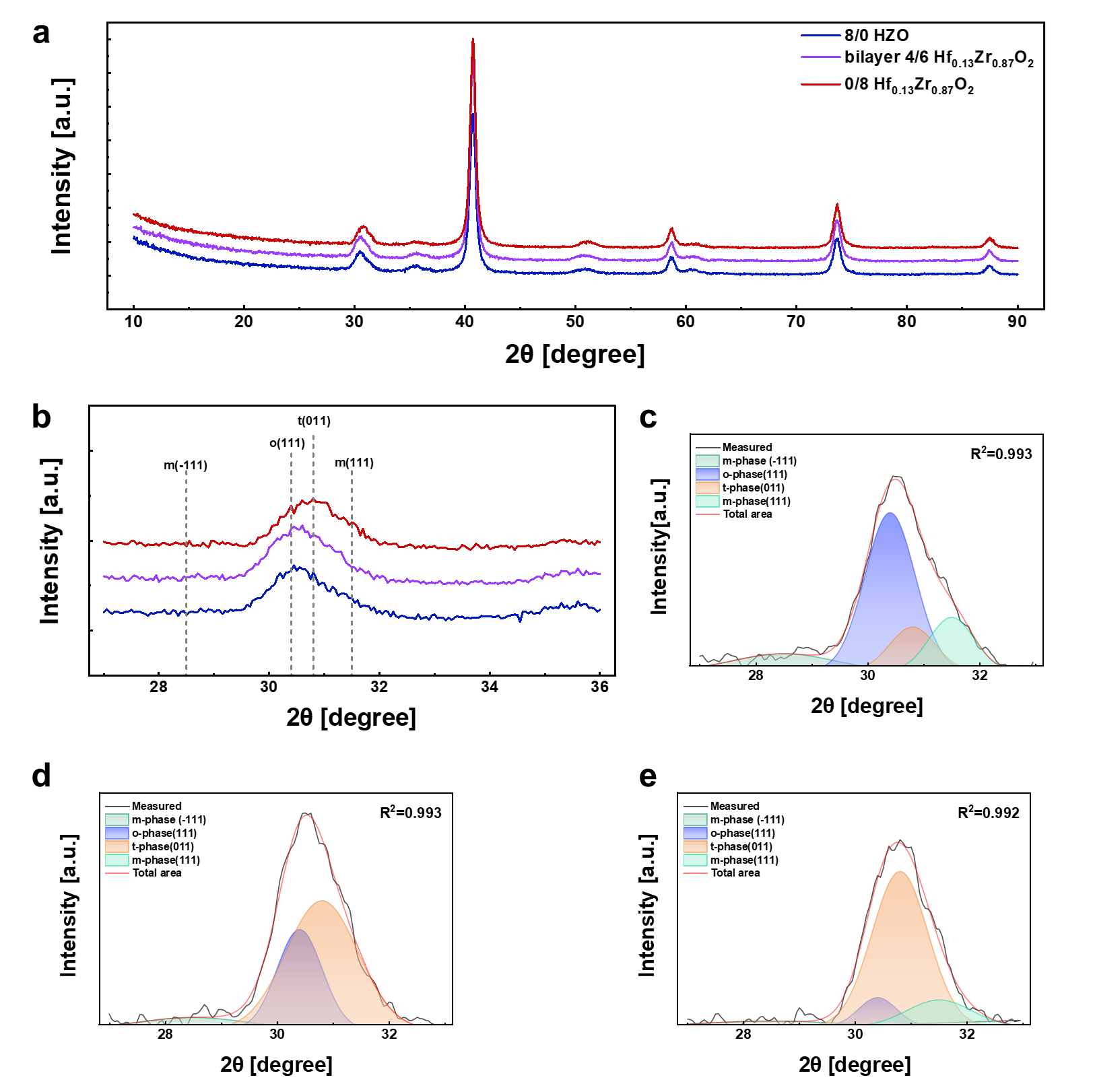


**Figure S6 |** (a) GI-XRD patterns of FE/AFE capacitors with different configurations film: 8/0 HZO, bilayer 4/6 Hf_0.13_Zr_0.87_O_2_, and 0/8 Hf_0.13_Zr_0.87_O_2_. (b) Enlarged 2θ region highlighting the representative diffraction peaks corresponding to the monoclinic (m), orthorhombic (o), and tetragonal (t) phases, with peak positions indicated by dashed lines. (c-e) Peak-deconvolution analysis of the m(–111), o(111), t(011), and m(111) components for (c) 8/0 FE-dominant HZO (d) 4/6 FE/AFE bilayer HZO (e) 0/8 AFE-dominant HZO films, showing the relative phase fractions obtained from fitting.

**
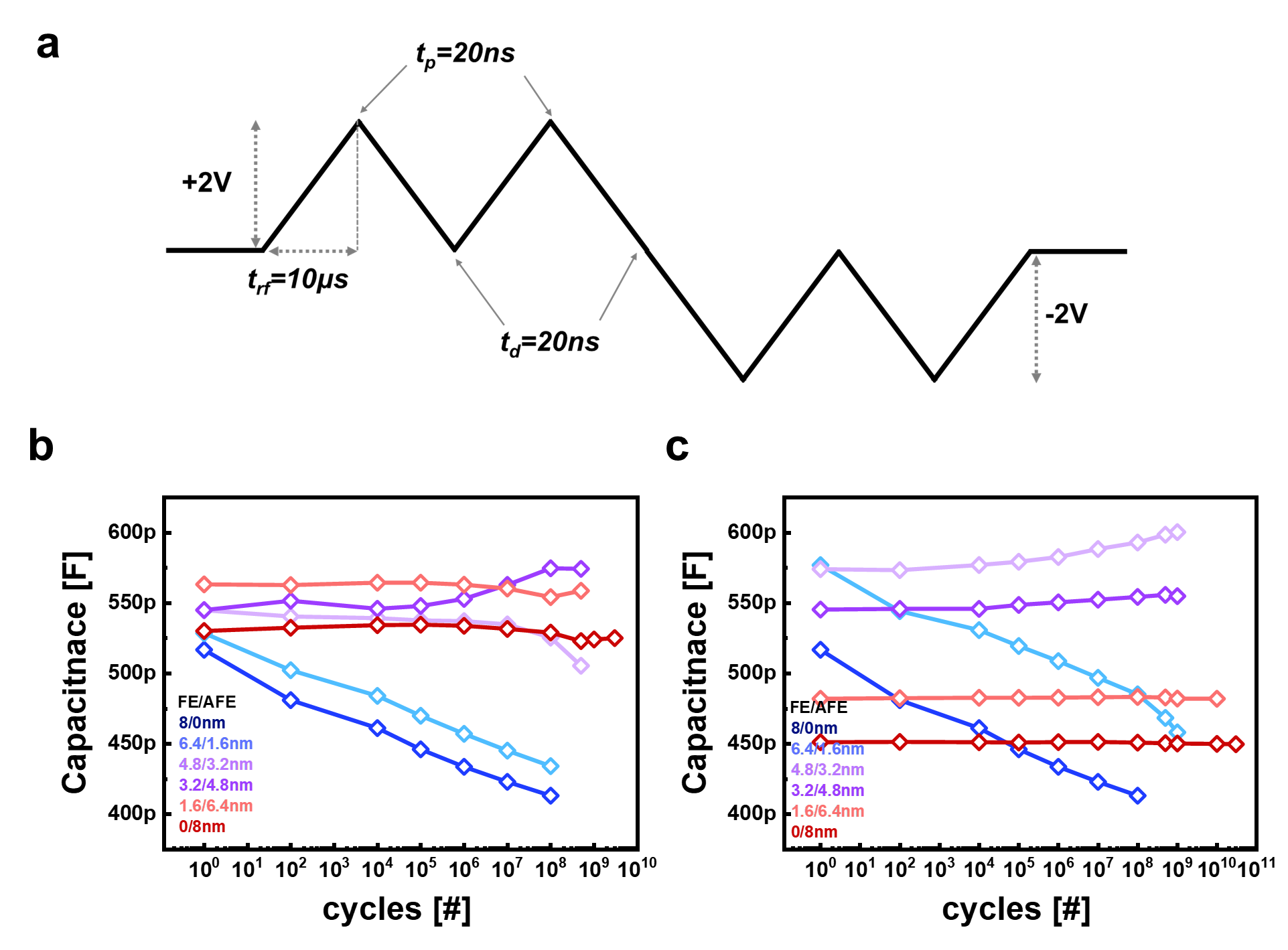
**

**Figure S7** **|**

(a) Schematic illustration of the triangular waveform (pulse voltage = ±2 V, rise/fall time (t_rf_) = 10 μs , peak duration (t_p_) =20 ns, delay time (t_d_) = 20ns) used for endurance measurements, corresponding to an equivalent cycling frequency of approximately 25 kHz. Capacitance endurance characteristics of FE/AFE bilayer capacitors under different thickness configurations with (b) Hf_0.25_Zr_0.75_O_2_ and (c) ZrO_2_ composition.


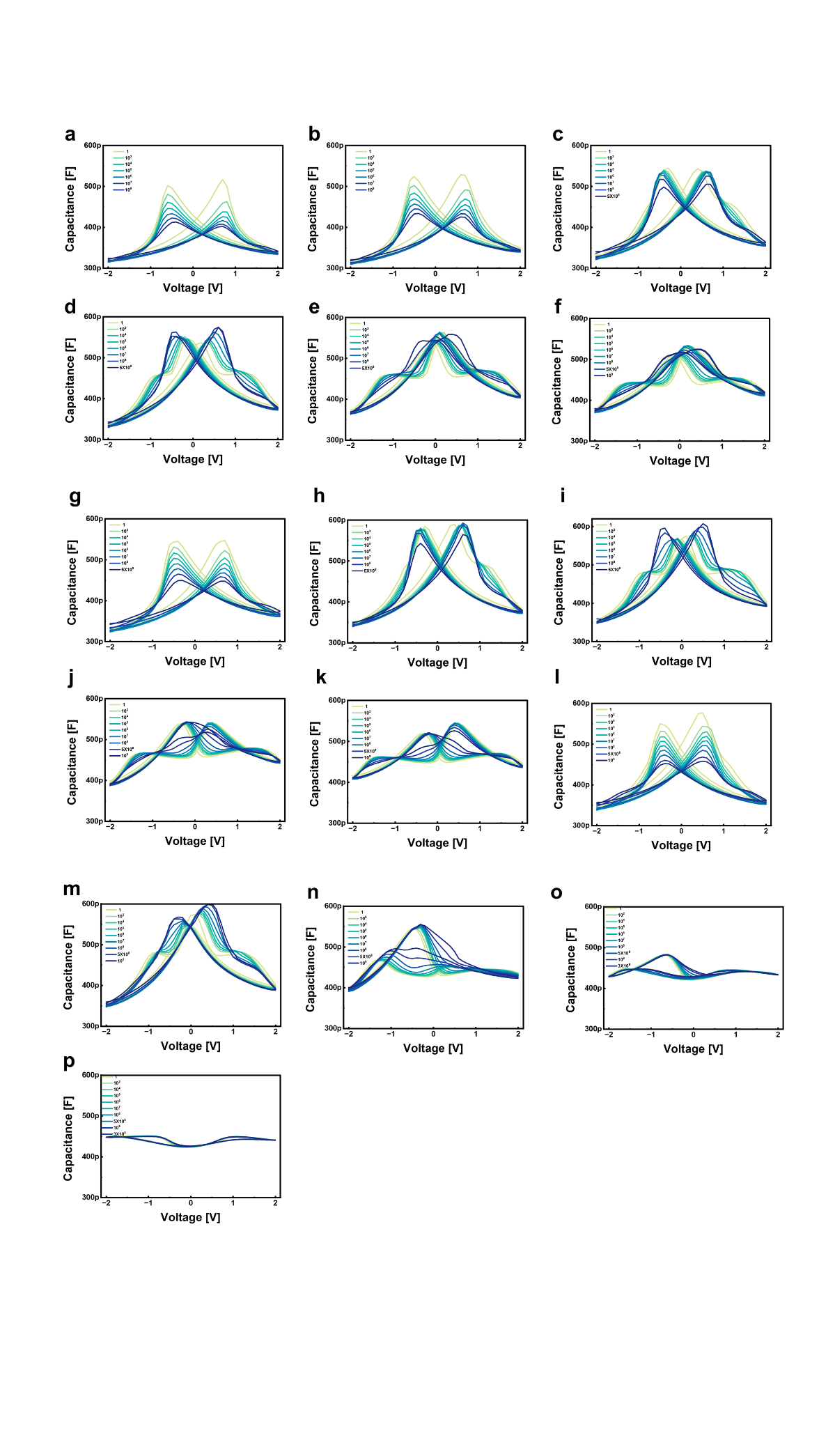


**Figure S8** **|** Capacitance–voltage (C–V) endurance curve of FE/AFE bilayer capacitors with different thickness configurations: (a) 8/0, (b) 6.4/1.6, (c) 4.8/3.2, (d) 3.2/4.8, (e) 1.6/6.4, and (f) 0/8 nm at of Hf_0.25_Zr_0.75_O_2_ Composition, (g) 6.4/1.6, (h) 4.8/3.2, (i) 3.2/4.8, (j) 1.6/6.4, and (k) 0/8 nm at of Hf_0.13_Zr_0.87_O_2_ Composition, (l) 6.4/1.6, (m) 4.8/3.2, (n) 3.2/4.8, (o) 1.6/6.4, and (p) 0/8 nm at of ZrO_2_ Composition.


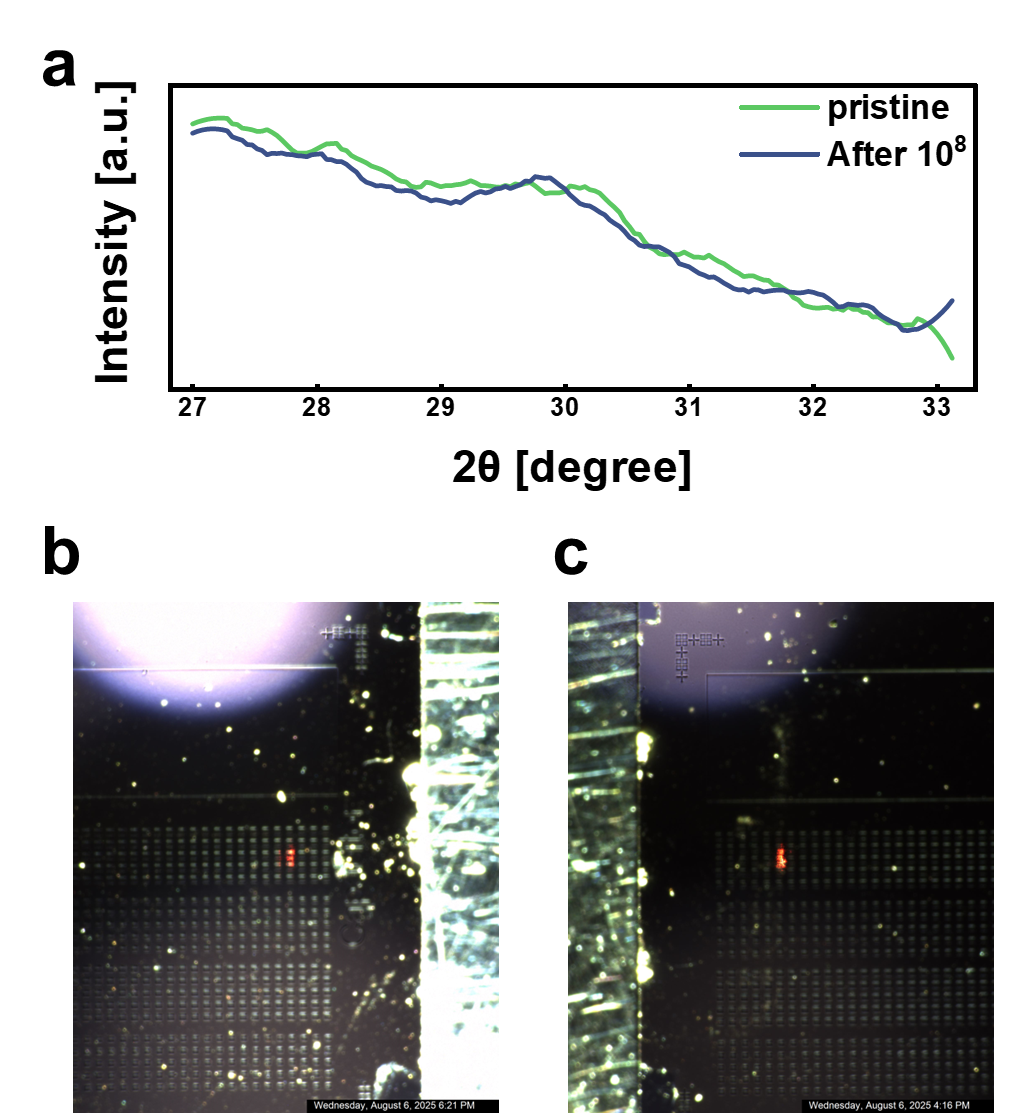


**Figure S9** **|** (a) Micro-XRD patterns of HZO films before cycling (pristine) and after 10⁸ cycles, (b) Pristine and (c) cycled region irradiated by micro-XRD beam.


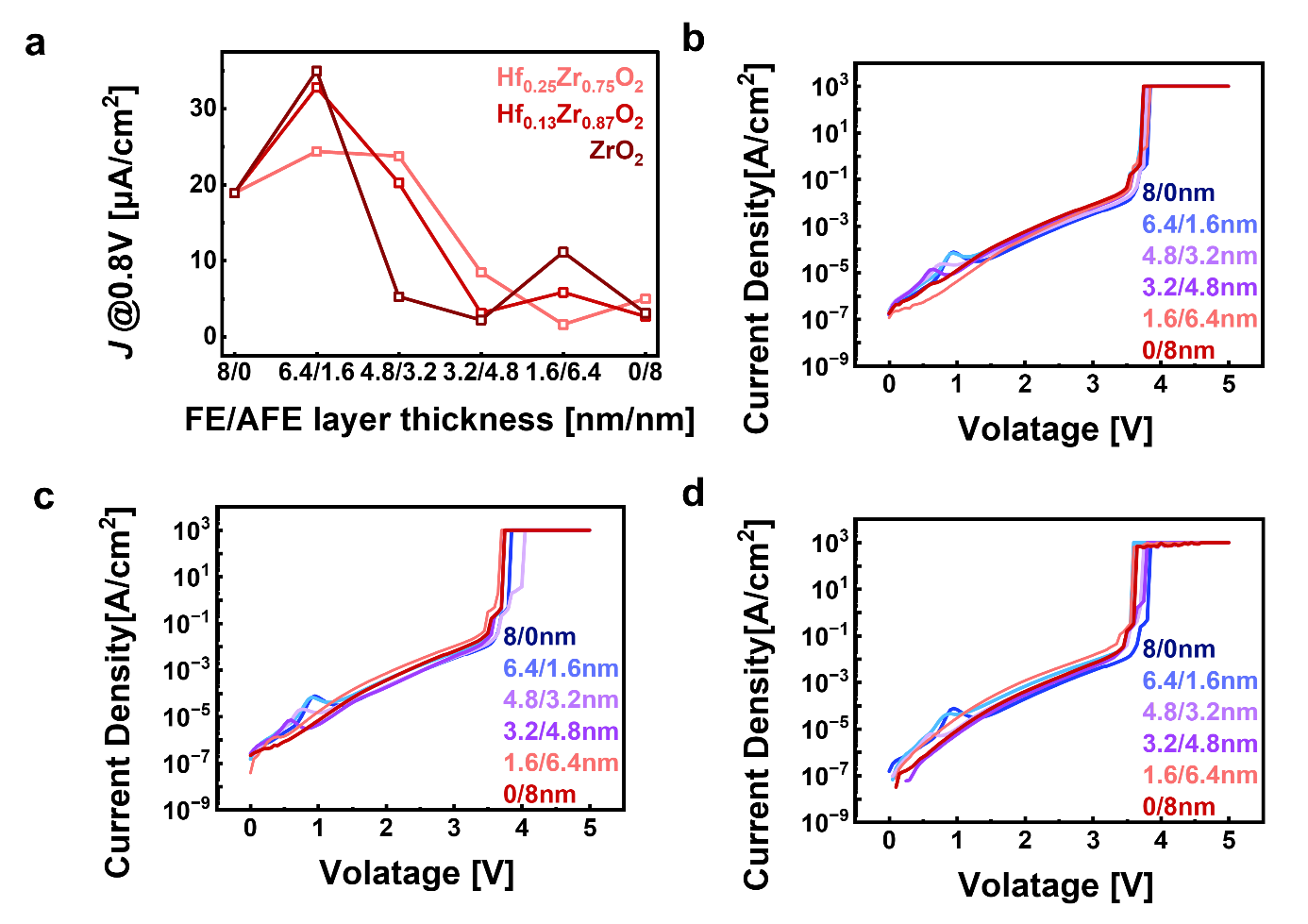


**Figure S10 |** (a) Extracted current density at 0.8 V for bilayer capacitors with AFE compositions of Hf_0.25_Zr_0.75_O_2,_ Hf_0.13_Zr_0.87_O_2_, and pure ZrO_2_.Current density–voltage (J–V) characteristics of FE/AFE bilayer capacitors measured from 0 to 5 V with different thickness configurations: (b) Hf_0.13_Zr_0.87_O_2_ (c) Hf_0.25_Zr_0.75_O_2_ and (d) ZrO_2_ composition.


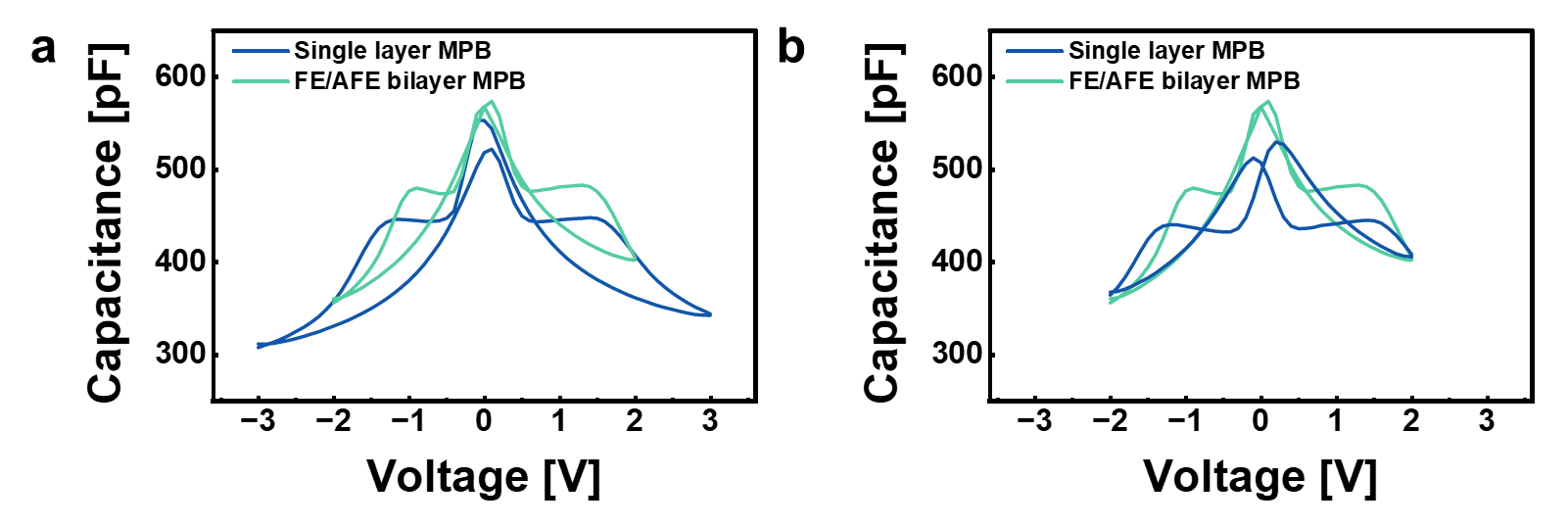


**Figure S11 |** (a) C–V characteristics of FE/AFE bilayer MPB (Hf_0.5_Zr_0.5_O_2_ 3.2 nm / Hf_0.13_Zr_0.87_O_2_ 4.8 nm) and single-layer MPB (Hf_0.33_Zr_0.67_O_2_ 8 nm), measured with sweep voltages of 2 V and 3 V, respectively. (b) C–V characteristics of FE/AFE bilayer MPB and single-layer MPB capacitors measured under the same sweep voltage of 2 V.
